# COVID‐19: a major cause of cachexia and sarcopenia?

**DOI:** 10.1002/jcsm.12589

**Published:** 2020-06-09

**Authors:** John E. Morley, Kamyar Kalantar‐Zadeh, Stefan D. Anker

**Affiliations:** ^1^ Division of Geriatric Medicine Saint Louis University School of Medicine St. Louis MO USA; ^2^ Division of Nephrology University of California Irvine CA USA; ^3^ Department of Cardiology (CVK), and Berlin Institute of Health Center for Regenerative Therapies German Centre for Cardiovascular Research Partner Site Berlin, Charité Universitätsmedizin, Berlin Germany

**Keywords:** COVID‐19, Cachexia, Sarcopenia, Coronavirus

Coronavirus disease (COVID‐19) has reached pandemic proportions. Two animal studies have shown that coronavirus‐2 causes weight loss in animals associated with an increase in inflammatory cytokines. In humans, COVID‐19 causes anorexia, weight loss and low albumin. While poorly studied, this suggests that severe COVID‐19 is associated with cachexia. The angiotensin converting enzyme 2 is the receptor for coronavirus‐2, and it occurs in skeletal muscle. Persons with COVID‐19 have myalgias and muscle loss. This coupled by bed rest and being ventilated can lead to severe sarcopenia during the recovery period following COVID‐19. Coronavirus 2 disease (COVID‐19) is a pandemic that swept around the world.^1^ The disease starts as a nasopharyngeal infection; it can sweep through the body infecting almost every organ (Figure 1). The coronavirus‐2 spikes protein, uses the angiotensin converter enzyme 2 (ACE2) receptor to bind to a cell resulting in fusion of the viral envelope to fuse with cell membrane and allows the viral genetic material to enter the cell.^2^ ACE2 receptors are present ubiquitously throughout the body resulting in a variety of tissue damages. It is important to recognize that many persons infected with coronavirus‐2 have no or minimal symptoms. Others develop severe disease. People at highest risk appear to be those with comorbidity, diabetes, hypertension, smokers and older individuals.^3^

**FIGURE 1:**
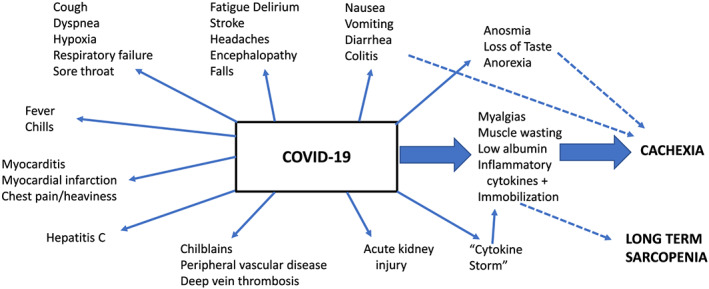
The effects of COVID‐19

Cachexia is defined by our society as ‘a complex metabolic syndrome associated with underlying illness and characterized by loss of muscle.’^4^ Its clinical features are weight loss, low albumin, anorexia, increased muscle protein breakdown and inflammation. Weight loss is a feature of COVID‐19 and was clearly demonstrated by the CNN television host who lost 13 pounds over 2 weeks while infected with COVID‐19. Both myalgias and muscle loss have been seen in COVID‐19.^5^ Muscle has an ACE2 receptor which might in part explain these effects. Persons with COVID‐19 also have hypoalbuminemia and elevated levels of C‐reactive protein and a number of inflammatory cytokines such a tumour necrosis alpha, interleukin‐1 and interleukin‐6.^6^, ^7^ A further cause of muscle loss and weakness is the immobilization seen in mechanically ventilated patients in the intensive care unit.^8^

Anorexia is a component of COVID‐19.^1^ This is, in part, due to the anosmia and loss of taste that occurs in COVID‐19, but is also secondary to the elevated levels of inflammatory cytokines, which are common causes of anorexia.^9^

When Syrian hamsters are injected with coronavirus‐2, they develop typical signs of COVID‐19 as well as weight loss.^10^, ^11^ This is associated with increases in interferonδ and tumour necrosis alpha. Mice infected with coronavirus‐2 had had significant weight loss which was reversed by a ribonucleoside analog.^12^

Sarcopenia is defined as the decreased muscular function in the presence of muscle loss.^13^ Primary sarcopenia is age related while secondary sarcopenia is when the sarcopenia is related to a chronic disease such as diabetes mellitus or chronic obstructive pulmonary disease.^14^ In older persons, the need for social isolation during the COVID‐19 pandemic has led to a decrease in daily physical activity which accelerates the loss of muscle strength and function. Persons with diagnosed COVID‐19 are also likely to have 2 or 3 weeks of decreased function resulting in secondary sarcopenia. Following COVID‐19, a number of persons have lung damage with hypoxemia. High‐altitude hypoxia leads to loss of fat‐free mass and physical disability.^15^

Persons who are physically isolated should be given recommendations to do daily exercises such as the ViviFrail graded exercise set (^16^ vivifrail.com). There is evidence that persons in hospital have better outcomes if they receive exercise therapy during hospitalization.^17^ There is evidence that persons with severe COVID‐19 need prolonged exercise therapy to prevent or reverse disability.^18^

Cachexia and sarcopenia are major causes of mortality and disability. Persons who survive cachexia often require long periods of rehabilitation. Similarly, persons who develop sarcopenia secondary to a stressful event often require lifetime exercise and nutrition therapy. We need more research into the incidence of cachexia in COVID‐19 and also more attention to rehabilitation during the recovery phase.^19^

## Conflict of interest

The authors declare there are no conflicts of interest.
